# Staffing and Capacity Planning for SARS-CoV-2 Monoclonal Antibody Infusion Facilities: A Performance Estimation Calculator Based on Discrete-Event Simulations

**DOI:** 10.3389/fpubh.2021.770039

**Published:** 2022-01-28

**Authors:** Çaglar Çaglayan, Jonathan Thornhill, Miles A. Stewart, Anastasia S. Lambrou, Donald Richardson, Kaitlin Rainwater-Lovett, Jeffrey D. Freeman, Tiffany Pfundt, John T. Redd

**Affiliations:** ^1^Asymmetric Operations Sector, Johns Hopkins University Applied Physics Laboratory, Laurel, MD, United States; ^2^Office of the Assistant Secretary for Preparedness and Response, United States Department of Health and Human Services, Washington, DC, United States

**Keywords:** coronavirus disease 2019 (COVID-19), capacity-planning, staffing, discrete-event simulation, monoclonal antibody treatment, decision-support tool, disaster preparedness and response

## Abstract

**Background:**

The COVID-19 pandemic has significantly stressed healthcare systems. The addition of monoclonal antibody (mAb) infusions, which prevent severe disease and reduce hospitalizations, to the repertoire of COVID-19 countermeasures offers the opportunity to reduce system stress but requires strategic planning and use of novel approaches. Our objective was to develop a web-based decision-support tool to help existing and future mAb infusion facilities make better and more informed staffing and capacity decisions.

**Materials and Methods:**

Using real-world observations from three medical centers operating with federal field team support, we developed a discrete-event simulation model and performed simulation experiments to assess performance of mAb infusion sites under different conditions.

**Results:**

162,000 scenarios were evaluated by simulations. Our analyses revealed that it was more effective to add check-in staff than to add additional nurses for middle-to-large size sites with ≥2 infusion nurses; that scheduled appointments performed better than walk-ins when patient load was not high; and that reducing infusion time was particularly impactful when load on resources was only slightly above manageable levels.

**Discussion:**

Physical capacity, check-in staff, and infusion time were as important as nurses for mAb sites. Health systems can effectively operate an infusion center under different conditions to provide mAb therapeutics even with relatively low investments in physical resources and staff.

**Conclusion:**

Simulations of mAb infusion sites were used to create a capacity planning tool to optimize resource utility and allocation in constrained pandemic conditions, and more efficiently treat COVID-19 patients at existing and future mAb infusion sites.

## Introduction: Background and Significance

Spreading rapidly, Coronavirus disease 2019 (COVID-19) became a global pandemic in early 2020 (1). Caused by severe acute respiratory syndrome coronavirus 2 (SARS-CoV-2), COVID-19 is a highly contagious disease that can result in mild to severe symptoms, hospitalization, need for intensive care or ventilator treatment, and mortality (2). By July 2021, SARS-CoV-2 had infected more than 33 million Americans, caused over 600,000 deaths in the United States (U.S.) (3), and killed more than 3,900,000 people worldwide (4).

In November 2020, the Food and Drug Administration issued emergency use authorizations for monoclonal antibody (mAb) monotherapy bamlanivimab and combination therapy casirivimab/imdevimab to treat COVID-19 among individuals at high risk for progressing to severe disease (5). These mAb treatments were shown to be effective for preventing progression of disease and COVID-19-associated hospitalizations (6, 7). Given that a significant number of individuals remain at risk for COVID-19 in the U.S. (8), mAb treatments will continue to be critical for saving lives and reducing COVID-19 morbidity and mortality (9, 10).

In the context of capacity and staffing shortages, mAb therapies are especially important to reduce burdens on the U.S. healthcare system. However, these treatments have administration challenges, including authorized use only in confirmed COVID-19 outpatients; the need to treat patients as early as possible; and the messaging required to introduce mAb products to providers and patients in an already-stressed system (11). Moreover, an outpatient mAb treatment requires the allocation of staff, physical space, equipment, and supplies, and involves several sequential treatment procedures necessitating synchronization. Thorough analysis and careful planning are needed to decrease barriers to implementation and ensure efficient service.

As of October 14, 2021, there are thousands of healthcare facilities in the U.S. that periodically receive and offer mAb treatments (12). In those healthcare facilities, mAb administrations occur across a wide variety of settings including hospitals, hospital-based infusion sites, emergency departments, urgent care clinics, and stand-alone infusion centers (13). These infusion sites have diverse physical capacity and staffing limitations, requirements, and service features, including differences in the patterns and volumes of daily patient demand, appointment scheduling regimes, service hours, service and performance targets, and state and local regulations. Accordingly, critical planning and resource allocation decisions for these mAb infusion sites must be made locally to ensure the best service quality and optimal performance. Developed upon the request of (and now administered by) the U.S. Assistant Secretary for Preparedness and Response (ASPR), the decision-support tool that we present in this paper serves this exact purpose by helping infusion sites tailor their capacity and staffing decisions, and has highly valuable utility in the face of a surge of COVID-19 cases and increased demand for mAb treatments fueled by the highly contagious Delta variant (14, 15).

The objective of this study was to create a capacity and staffing planning tool to support the implementation of mAb drug products at U.S. healthcare facilities. Taking a simulation approach, we developed a web-based calculator to investigate the operational performance of mAb infusion sites as a function of staffing, capacity, and other key factors. The decision-support tool can be used to identify bottlenecks in the mAb infusion process, and help decision-makers make informed resource allocation decisions for mAb treatment service.

## Materials and Methods

Discrete-event simulation (DES) experiments were conducted for 162,000 alternative scenarios considering different staffing and capacity levels, scheduling protocols, patient demand, facility service hours, and infusion durations. To inform the model structure and collect data for simulation experiments, the mAb treatment process was observed at three U.S. medical centers implementing mAb infusions in collaboration with Disaster Medical Assistance Teams deployed by the US Department of Health and Human Services under the direction of the Assistant Secretary for Preparedness and Response's National Disaster Medical System. To make simulation results accessible, a web-based decision-support tool was developed, which displays estimated performance outputs for the scenarios associated with user-selected inputs. These inputs are described in Web-based decision-support tool: mAb infusion process calculator. The DES model and simulation experiments were programmed using Matlab version R2020a.

### Patient Arrival Process

Infusion facilities either had walk-in encounters, where patients were admitted to check-in area on a first-come-first-served basis, or utilized scheduled appointments of three different types: (i) block, (ii) spread-out, and (iii) mixed (Table 1). A block schedule uses only a few scheduling points (e.g., 9 AM, 1 PM, 4 PM) and books a batch of patients for each time block. Spread-out scheduling uses numerous scheduling points and books a small number of patients for the same time point to more evenly distribute patient load on resources. Lastly, mixed scheduling strikes a balance between block and spread-out scheduling by being more dispersed than block and more condensed than spread-out scheduling. Delays and early arrivals were accounted for via a delay function that adjusts patient arrival times by adding (or subtracting) some random amount of time to (from) appointment times.

**Table 1:** Different appointment types considered in our analysis.

**Appointment type**	**Brief description**	**An example with 15 patients**
Walk-in only	Unscheduled appointments with random arrivals	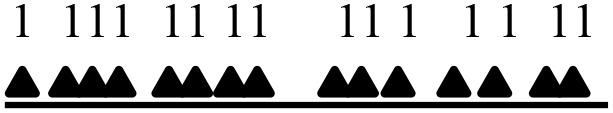
Scheduled only - Block	Schedules with few appointment blocks, where a large group of patients are scheduled for each block	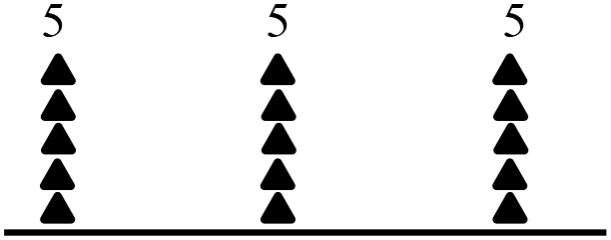
Scheduled only - Spread-Out	Schedules with a large number of appointment times throughout the operating hours, where only a single or a few individual(s) are scheduled for each	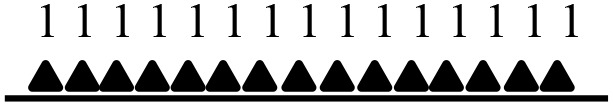
Scheduled only -Mixed	Schedules with relatively few appointment blocks, where appointment times are dispersed within each block to balance patient load	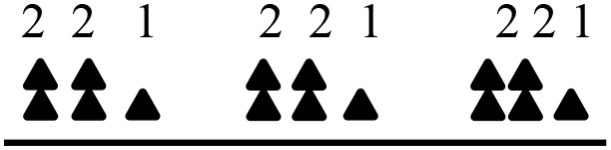

### Monoclonal Antibody Treatment Process

**Figure 1:**
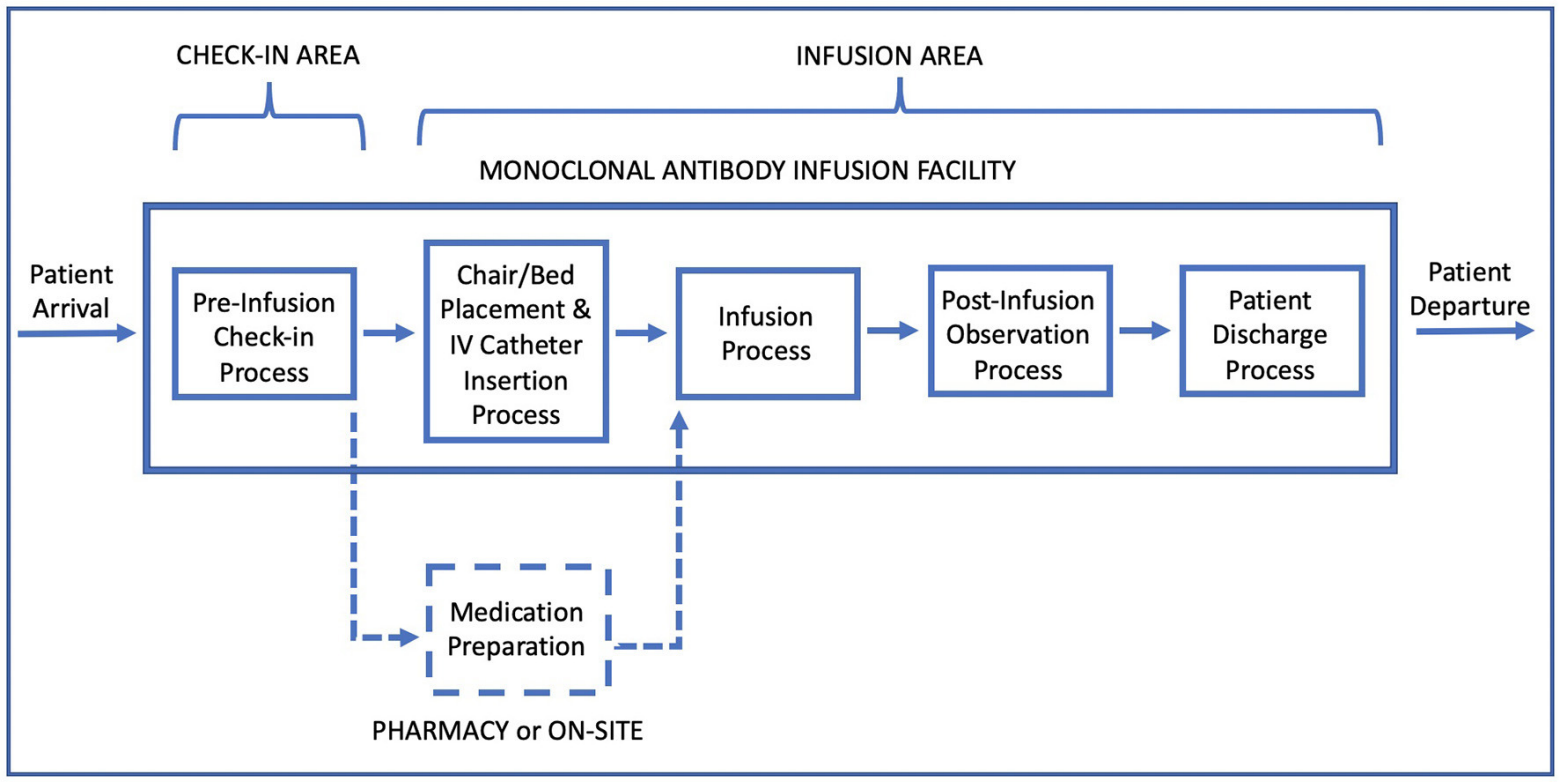
Patient flow diagram in a monoclonal antibody infusion facility.

The mAb treatment process in the decision-support tool starts with pre-infusion check-in (Figure 1). There are no capacity restrictions in the check-in area, as arriving patients are directed to wait outside when this area is fully occupied. Each check-in area staff serves a single patient at a time and performs clerical and clinical duties such as obtaining patient information and signatures for consent forms, verifying patient identification and health insurance, providing information about the treatment, and checking vitals. At the end of this process, clinical staff in walk-in facilities inform the pharmacy to initiate medication preparation. This step is bypassed for scheduled appointments, as medications are assumed to be prepared in advance for scheduled visits.

Following the waiting time after check-in, patients enter the infusion area and are directed to a treatment chair/bed. Consisting of nurses and allied health professionals (e.g., paramedics), the medical team in the infusion area prepare patients for infusion by conducting a medical examination, and inserting a peripheral intravenous (IV) catheter for drug administration. “Chair/Bed placement and IV catheter insertion” is followed by the infusion process. Infusion therapy requires a fixed amount of time, depending on medication, and is delayed when there is no medication readily available or if the medical care team is busy attending other patients.

The infusion process is followed by an observation period, during which patients remain seated and are monitored. Post-infusion observation period takes an hour but might be prolonged by concerns about a patient's health status. Following the observation, the IV is removed and each patient goes through a discharge process, waiting for the final paperwork and the discharge consent from the physician in charge. Subsequently, patients depart the mAb infusion facility.

### Data Sources, Data Collection, and Simulation Parameters

The parameters and probability distributions used in simulation experiments reflect the data collected during observation of three U.S. medical centers implementing mAb infusions (Table 2). These parameters correspond to service durations and do not include preceding or succeeding waiting times. Data and descriptions for the generation of scheduled and walk-in arrivals and delays during the observation process are provided in the Supplementary Material—Appendix A.

**Table 2:** Probability distributions and parameters for mAb treatment sub-processes.

**Process**	**Probability distribution**	**Parameters (minutes)**
Pre-infusion check-in	Exponential	Mean = 20
Chair placement & IV catheter	Exponential	Mean = 10
Infusion	Deterministic	Duration = 20, 30, or 60
Post-infusion observation	Deterministic	Duration = 60
Discharge	Normal	Mean = 5, Standard Deviation = 1
Medication preparation	Uniform, NA – if scheduled appt.	Minimum = 15, Maximum = 30 NA – if scheduled appointments

### Discrete-Event Simulation (DES) Model

A simulation model was developed to analyze the COVID-19 mAb infusion therapy process and assess the performance of mAb infusion sites under various scenarios. The model inputs and outputs were identified based on our discussions with clinical experts and partner mAb sites. Model inputs were (i) treatment bed/chair capacity, (ii) type of appointments, (iii) service hours, (iv) daily patient demand, (v) infusion duration, and (vi) staffing levels at check-in and infusion areas (Table 3). Model outputs were (i) average patient length of stay (LoS) with 95% confidence intervals, (ii) number and percentage of patients treated during service hours, and (iii) percentages of patients treated within 3 and 4 h.

**Table 3:** Model inputs for discrete event simulation model.

**Model variables**	**Alternative input values**	**Number of alternatives**
Physical capacity – number of treatment beds/chairs	3, 5, 6, 9, 10, 12, 15, 20, 30	9
Appointment type	Walk-in, Scheduled – Block, Scheduled – Mixed, Scheduled – Spread-Out	4
Operating hours	6, 8, 10, 12, 24	5
Daily patient demand	10, 15, 20, 25, 30	5
Infusion duration	20, 30, 60 minutes	3
Check-in area staffing levels	1, 2, 3	3
Infusion area staffing levels (Nurse)	1, 2, 3, 4	4
Infusion area staffing levels (Allied Health Professional)	0, 1, 2, 3, 4	5

Inputs for staffing levels were concentrated on the number of nurses and allied health professionals. Other roles, such as physicians or pharmacists, are also critical for infusion facilities, but were not observed to directly limit the capacity and patient flow. Based on on-site observations, a nurse was assumed to deliver concurrent care for up to five patients, whereas an allied health professional was assumed to simultaneously care for up to three patients. Model assumptions required at least one nurse to be present in infusion area. The type of clinical staff was assumed not to impact check-in tasks, where a single employee serves one patient at a time.

The simulated events for mAb infusion process were (i) patient arrivals, (ii) check-in service completion, (iii) service completion for chair/bed placement and IV catheter insertion, (iv) completion of infusion therapy, (v) end of post-infusion observation, (vi) patient departure following discharge process, and (vii) closure of the infusion clinic. Walk-in clinics required an additional event of medication preparation since they do not start preparing infusion medication before patient arrival and check-in. The model structure and status updates performed for these events are described in the Supplementary Material—Appendix B.

In this study, our goal was to focus on provision of efficient treatment. Variables that may affect provision of treatment include, but are not limited to, the effectiveness of treatments, human and physical resources of a treatment facility, patient load on an individual treatment site or the general healthcare delivery system, and the duration of sequential sub-processes that constitute the overall treatment process. Accordingly, we concentrated on capturing these factors in our discrete event simulation model, rather than the ones that are considered in epidemiology models for the spread of an infectious disease over time, as our focus was on the treatment process and our objective was to develop a decision-support tool to help mAb infusion sites with their staffing and capacity planning decisions.

### Model Verification and Validation

A verification analysis was conducted to confirm that the simulation model was consistent with the mAb therapy process and experiments were correctly implemented. Examining 162,000 simulated scenarios, we observed that patient LoS and other output metrics either improved or remained constant but never worsened when staffing or capacity levels were increased while other inputs were kept constant. Similarly, all output metrics improved, though not at the same magnitude, when infusion time was reduced from 60 to 30 min and from 30 to 20 min. Finally, all output measures in 40,500 walk-in scenarios worsened when medication preparation time range was increased from 15–30 to 30–60 min. These verification analyses confirmed logical outcomes and that the simulation experiments were performed correctly.

Since mAb infusion for COVID-19 is a novel therapy, there are insufficient data to perform a comprehensive validation analysis. However, the accuracy of the generated estimates was assessed using expert face validation. Several feedback and pilot sessions were conducted with partner mAb sites and experts were asked to validate estimates for performance metrics. Using the web-based decision-support tool, they evaluated multiple different scenarios, and affirmed that estimated outputs were in line with their observations and expectations.

### Web-Based Decision-Support Tool: mAb Infusion Process Calculator

**Figure 2:**
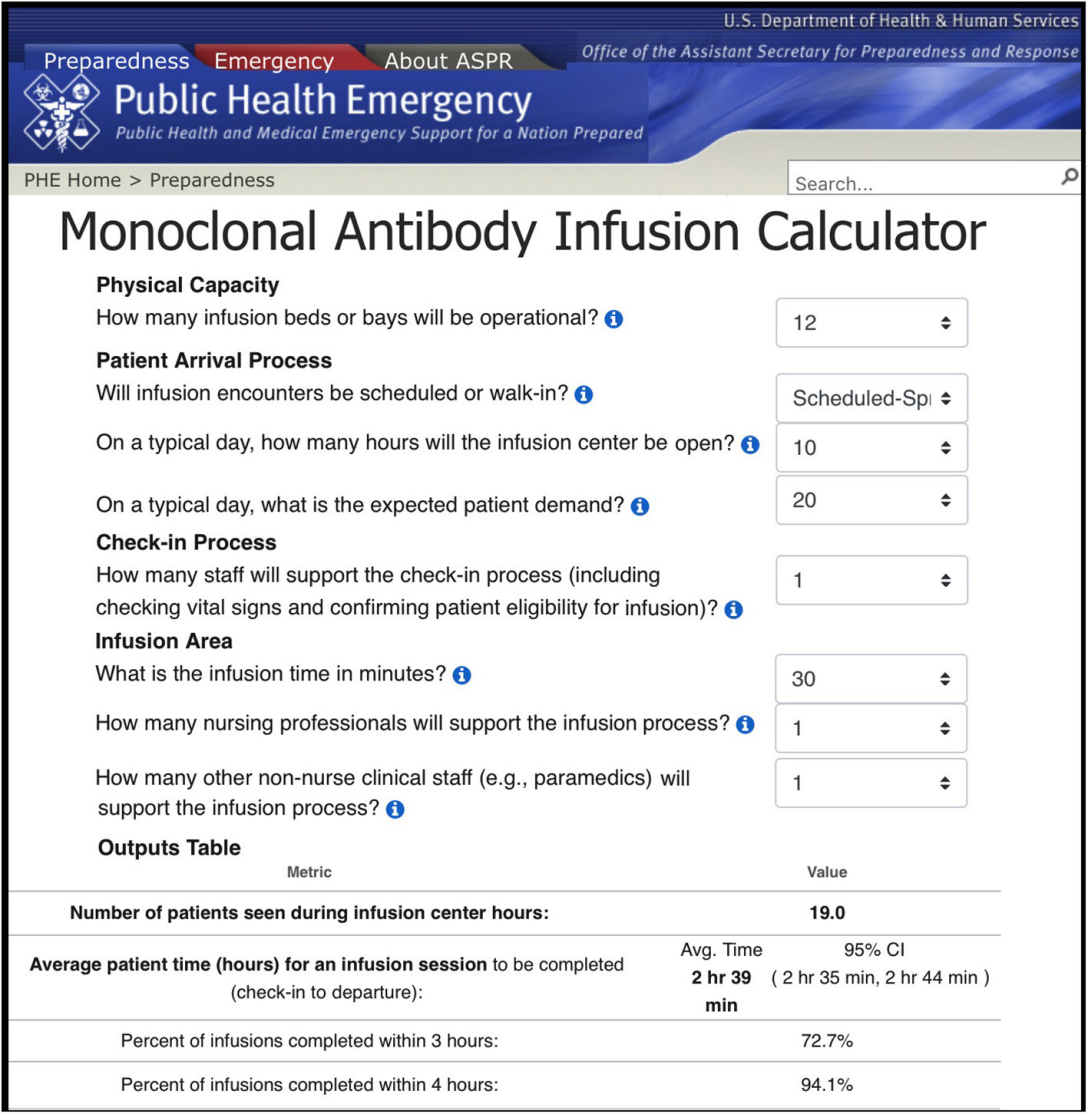
User Interface of the web-based mAb infusion process calculator (https://www.phe.gov/Preparedness/Pages/mabcalctool.aspx).

Utilizing simulation results, a web-based calculator (Figure 2) was created to assist capacity planning and resource allocation decisions for mAb infusion sites. At the website, users provide inputs for the main variables of a mAb infusion process (Table 3) and the outputs of the corresponding scenario are displayed to the user. There are 162,000 scenarios that users can investigate. All simulation experiments were performed in advance and saved on the website to provide instant feedback to users.

**Figure 3:**
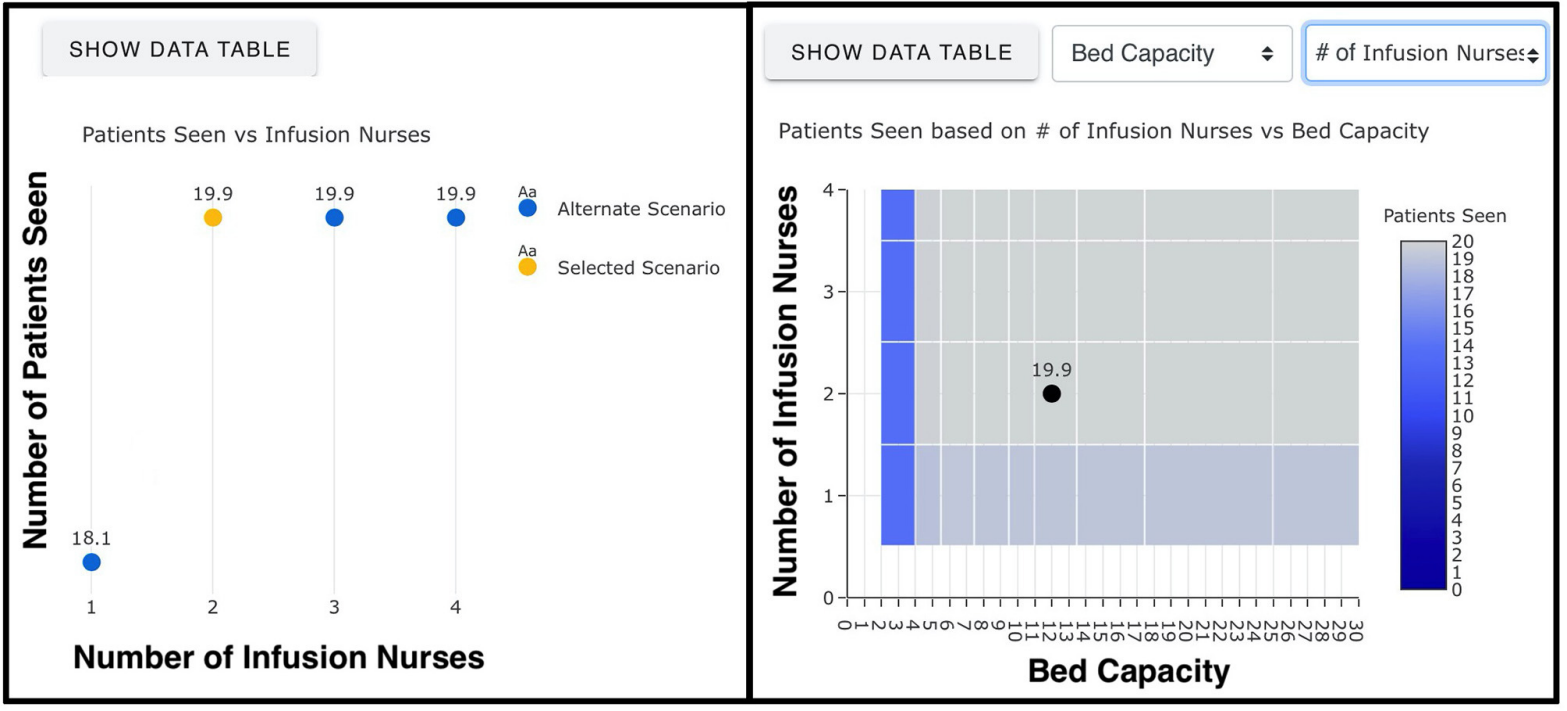
Graphs generated by the web-based mAb infusion process calculator (https://www.phe.gov/Preparedness/Pages/mabcalctool.aspx).

In addition to simulation outputs, the web-based calculator also displays two plots for each selected scenario (Figure 3). The first plot depicts the impact of increasing or decreasing the number of infusion nurses on the total number of patients that can be served throughout a day. The second plot shows the effect of any two variables chosen by the user (e.g., number of infusion nurses and bed capacity) on the number of patients treated. Together, the graphs help users assess the value of changing staffing levels for a given scenario, examine how added resources lead to improvements or diminishing returns, and identify the bottlenecks of the simulated scenario. Further, these graphs could be used to determine the minimum number of resources required to meet desired targets in terms of daily patient throughput.

As a programming language, Matlab version R2020a was employed to develop and code the simulation model and perform the simulation experiments. Twenty virtual machines, belonging to the Johns Hopkins University Applied Physics Laboratory, were utilized to expedite simulation experiments and processing of 162,000 scenarios. To translate the simulation results into a web-based calculator, a single-page application (SPA), the current standard for creating modern web applications, was produced. The use of SPA enabled the online calculator to be compiled into a simple HTML/JavaScript bundle. This bundle contained the outputs of the 162,000 scenarios, embedded into a page or run by a basic web server without the need for additional computational capabilities. Overall, the core frameworks used to develop the web-based platform were *Vue.js, Vuetify, Plotly.js*, and *Bootstrap*. In particular, the JavaScript framework “Vue.js” was used jointly with “Vuetify” to construct user interfaces and SPA, the data visualization library “Plotly.js” was utilized to create two interactive graphs (Figure 3) for each scenario, and “Bootstrap” was employed to enforce a uniform appearance to the website in terms of color, size, font and layout.

## Results

A total number of 162,000 different scenarios were evaluated by simulation experiments. Each infusion duration considered in the analysis (namely, 20-, 30-, and 60-min) corresponded to 54,000 different cases, where walk-in scenarios and different scheduling protocols each made up one fourth (i.e., 13,500) of these cases (Supplementary Material—Appendix C). All results can be accessed via the web-based calculator hosted at https://www.phe.gov/emergency/mAbs-calculator/Pages/default.aspx.

Based on 162,000 scenarios considered, various analyses can be performed to generate insights about different aspects of the mAb infusion process. To demonstrate the utility of the web-based calculator and shed light onto important planning and research questions for mAb infusion sites, we focused on four main areas: (i) the impact of scheduling on performance metrics, (ii) the effect of infusion duration on patient LoS, (iii) the role of medication preparation duration for walk-in encounters, and (iv) when and where to add additional staff to improve overall performance.

### Impact of Scheduling on Performance Metrics

To assess the impact of scheduling, we compared 13,500 walk-in scenarios with 60-min infusion times to their 13,500 spread-out scheduling counterparts. Excluding 3,457 scenarios having <10 min gap in LoS difference, we analyzed 10,043 scenarios with a non-negligible difference (i.e., ≥10 min gap) between LoS durations when spread-out scheduling was compared to walk-ins. Among these 10,043 scenarios, where the LoS difference were non-negligible, scheduled appointments had shorter LoS averages in 7,286 (72.5%) scenarios, and walk-in clinics achieved lower patient LoS in 2,757 (27.5%) cases.

Our analysis revealed that *traffic intensity*, a measure of the average occupancy of a service area, could play a key role in identifying when scheduling is the most and least beneficial. Traffic intensity estimates the average patient load on each resource (treatment bed or staff member) per hour by multiplying mean service duration (e.g., check-in time) with the average number of arrivals to the work station of interest per hour divided by the maximum resource capacity (beds or staff) available there. We observed that when the traffic intensity level was low to medium in infusion and check-in areas, indicating that the patient load on the mAb infusion site was manageable, scheduling yielded better outcomes and was an effective means for providing timely mAb treatments. In particular, there were 7,916 scenarios when the traffic intensity of the check-in process was <2.94 (i.e., medium), and scheduling resulted in lower LoS in 7,247 (91.5%) of these cases. Similarly, LoS averages were shorter with scheduling in 7,068 (90.2%) out of 7,839 scenarios when the traffic intensity in the infusion area was below 0.35 (i.e., low). However, scheduling had a limited impact on reducing LoS when the traffic intensity was high in either service area (Table 4). Compared to walk-in clinics, implementation of scheduled appointments achieved no improvement on LoS durations when traffic intensity was ≥ 3.86 in the check-in and ≥ 0.43 in the infusion area.

**Table 4:** Number of scenarios with shorter LoS under walk-ins and spread-out schedules.

**Location: check-in area**		**Total number of scenarios**	**Number of scenarios with shorter patient LoS**		**Location: Infusion area**		**Total number of scenarios**	**Number of scenarios with shorter patient LoS**	
**Metric** **=** **TI**	**Cut-off (CO)**	**TI** **<** **CO**	**Walk-in**	**Spread-out**	**Metric** **=** **TI**	**Cut-off (CO)**	**TI** **<** **CO**	**Walk-in**	**Spread-out**
**Check-in area**	0.64	1080	0	1080	**Infusion area**	0.10	2491	3	2488
**traffic intensity (TI)**	0.87	2160	0	2160	**traffic intensity (TI)**	0.18	5218	109	5109
	1.10	3220	0	3220		0.26	6888	354	6534
	1.33	4624	20	4604		0.35	7839	771	7068
	1.56	5156	80	5076		0.43	8490	1204	7286
	1.79	5886	246	5640		0.51	8903	1617	7286
	2.02	6500	367	6133		0.59	9149	1863	7286
	2.25	6834	435	6399		0.68	9565	2279	7286
	2.48	6834	435	6399		0.76	9739	2453	7286
	2.71	7916	907	7009		0.84	9918	2632	7286
	2.94	7916	907	7009		0.93	9918	2632	7286
	3.17	8602	1355	7247		1.01	9983	2697	7286
	3.40	8996	1749	7247		1.09	10023	2737	7286
	3.63	8996	1749	7247		1.18	10043	2757	7286
	3.86	9282	1996	7286		1.26	10043	2757	7286
	4.09	9282	1996	7286		1.34	10043	2757	7286
	4.32	9717	2431	7286		1.42	10043	2757	7286
	4.55	9717	2431	7286		1.51	10043	2757	7286
	4.78	9717	2431	7286		1.59	10043	2757	7286
	5.00	10043	2757	7286		1.67	10043	2757	7286

*CO,Cut-off value for traffic intensity levels; Traffic Intensity (TI),Average patient load over a single unit of limiting resource (staff or bed) per hour*.

### Effect of Infusion Time on Patient Length of Stay

**Figure 4:**
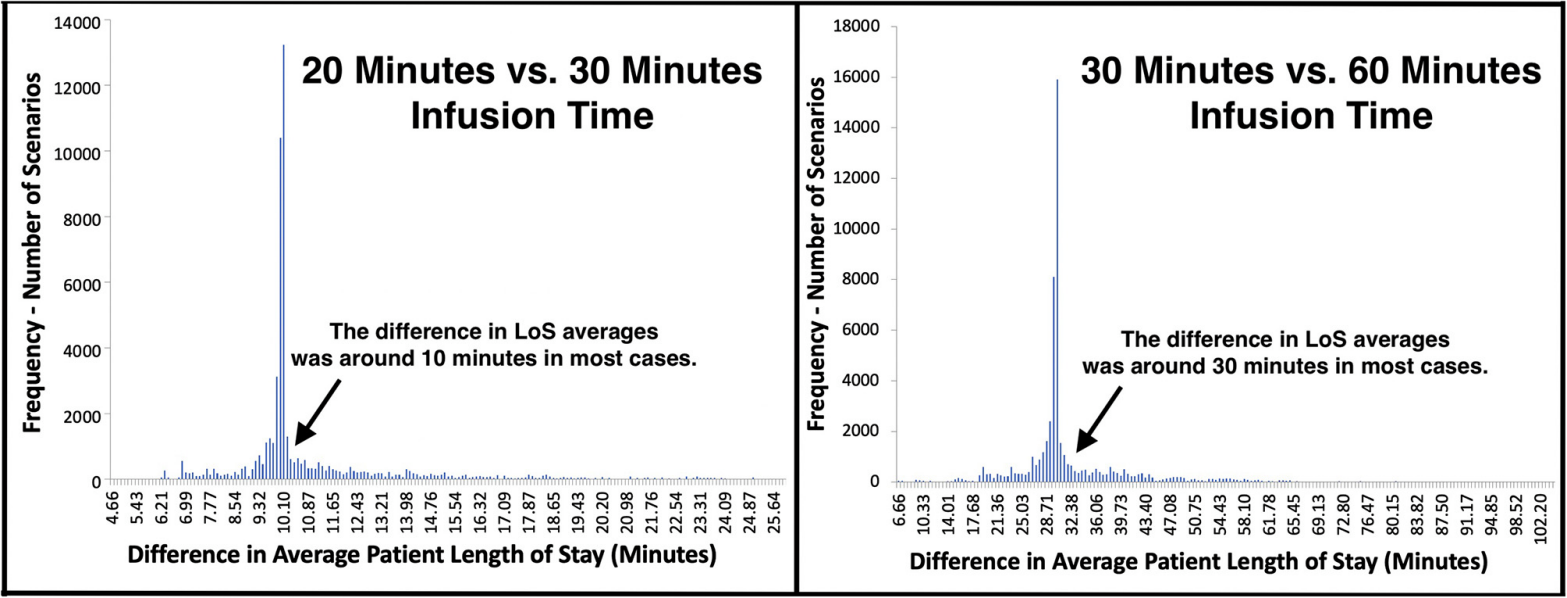
Distribution of LoS difference when different infusion times were compared.

Infusion times ranged from approximately 20–60 min. To investigate the effect of infusion time on patient LoS, we compared the average LoS outputs of 54,000 scenarios that had 30-min infusion time to their 108,000 counterparts with 20- and 60-min-long infusions. The distributions of LoS difference when 30-min infusions compared to other two alternatives are presented in Figure 4.

The differences in LoS averages centered around the amount of change in infusion times, which was a 10-min decrease and a 30-min increase for 20- and 60-min infusions, respectively (Figure 4). Yet, noticeably higher/lower changes in patient LoS were observed for a fair number of comparisons. In particular, 4,000 (7.4%) scenarios achieved > 45 min reduction in LoS when infusions took 30 min instead of 60 min. Similarly, 4,530 (8.4%) scenarios had >15 min difference in LoS when 20-min infusions were compared to 30 min infusions.

For the comparisons having significantly higher/lower change in LoS, queueing theory concepts “traffic intensity,” measuring the average occupancy of a service system, and “stability,” indicating whether its traffic intensity is at manageable levels or not, were helpful to explain this phenomenon (16, 17). The improvements observed in LoS were noticeably higher when reducing infusion duration led an unstable infusion site to experience manageable traffic intensity and become stable. This was because, by achieving stability, the whole service system functioned more efficiently, waiting times between consecutive services were reduced, and as a result, total reductions in LoS were higher than the change in infusion time. Similarly, the performance of a stable infusion site significantly worsened following an increase in infusion time when this change caused its traffic intensity to rise above manageable levels.

### Role of Medication Preparation Duration for Walk-In mAb Clinics

**Figure 5:**
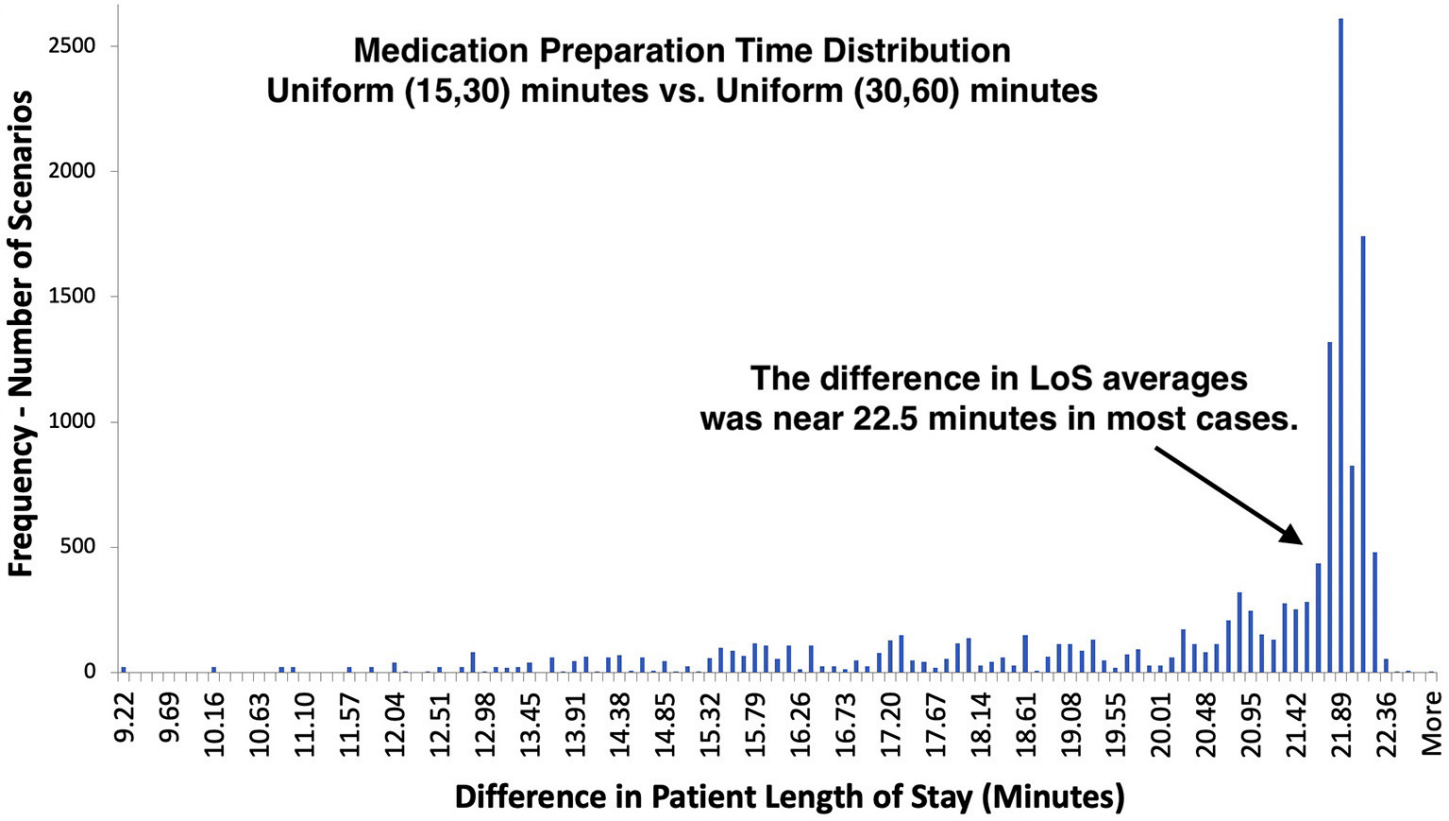
Distribution of LoS difference under different medication preparation times – comparison of 13,500 walk-in scenarios with 60 min infusion time.

At walk-in clinics, the preparation of mAb solution was performed after patient check-in rather than beforehand. In general, this might cause delays in infusion therapy. To quantify the effect of medication preparation on patient LoS, we conducted additional experiments, where medications were prepared within 30–60 min, and compared them to the baseline scenarios of 15–30-min-long medication preparation time. The difference between LoS averages mostly accumulated near 22.5 min, which was the average difference between 15–30 (mean time 22.5) and 30–60 (mean time 45.0) min preparation times (Figure 5). Changing the duration made less impact on LoS when physical capacity was low (i.e., number of beds ≤ 6) or patient load was high (i.e., ≥15 patients in ≤ 12 service hours). For those infusion sites, increasing bed capacity or staffing could be more beneficial than preparing infusion medications faster.

### Staff Adjustments to Improve Overall Performance of a mAb Infusion Site

**Figure 6:**
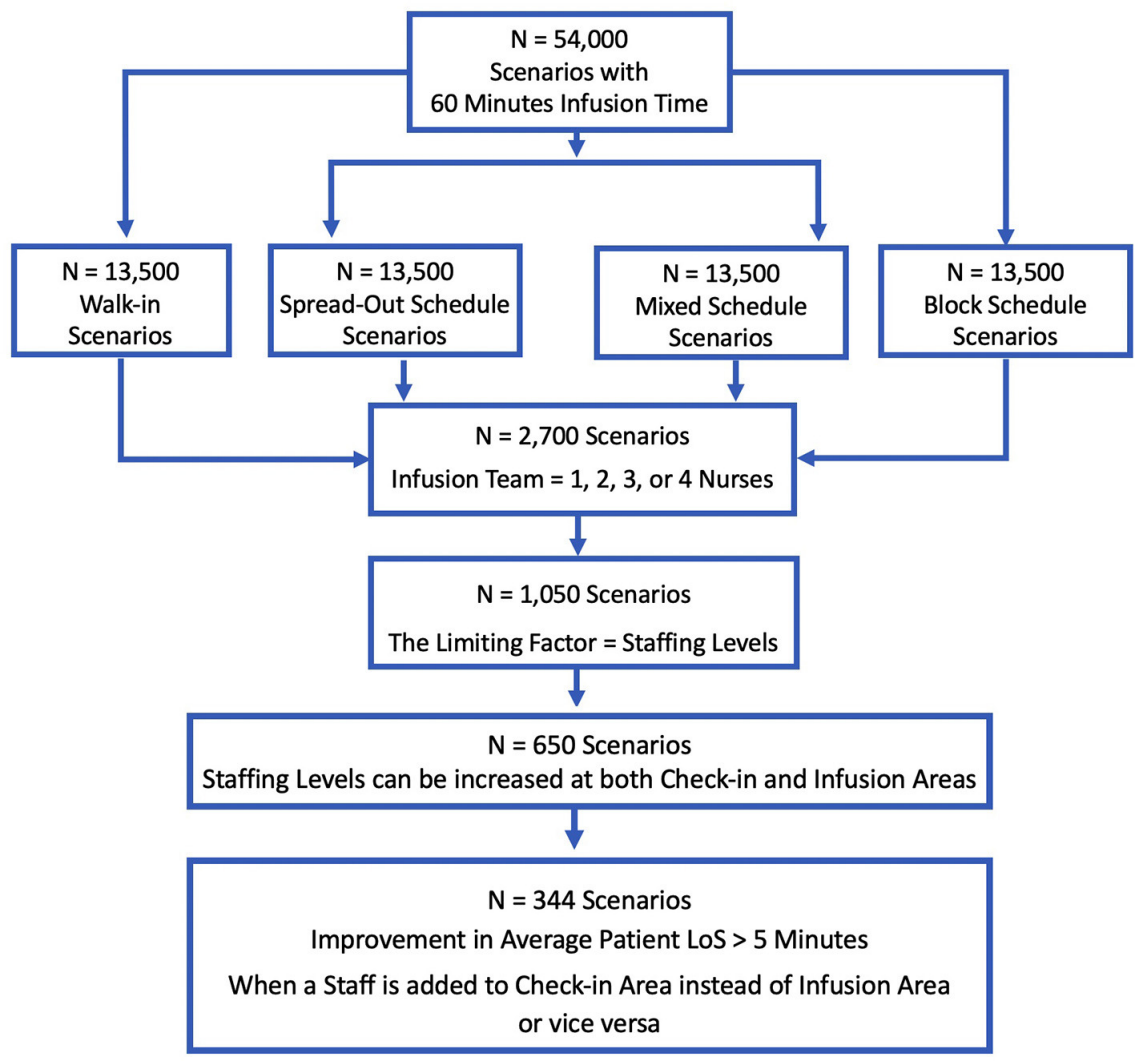
CONSORT diagram - the number of scenarios included in staff adjustment analysis.

Simulated scenarios with 60-min infusion times (*n* = 54,000) were examined separately for each scheduling type (*n*= 13,500) to assess the impact of adding staff members under different roles (Figure 6). To simplify the analysis, the scenarios, where infusion teams consisted of only nurses (*n* = 2,700), were considered. The cases for which the limiting factor was physical capacity rather than staffing were excluded. Consequently, a total of 650 scenarios were identified, for which staffing levels in check-in and infusion areas could both be increased. For these 650 scenarios, a staff was exclusively added to only one area and then, LoS averages were compared.

In 344 scenarios, the reduction in LoS achieved by adding a staff to an area were >5 min shorter than the other alternative (Figure 6). In 212 (62%) scenarios, adding an infusion nurse resulted in a larger decrease to LoS (mean 34 min). In the remaining 132 (38%) scenarios, where adding a check-in staff member was more beneficial (mean 24 min), physical capacity was ≥12 beds, the number of infusion area nurses was ≥2, and check-in staff were always originally fewer. These similarities in 132 scenarios suggest that, for medium-to-large size infusion sites having more than 10 or 11 beds, providing support to check-in area might be more beneficial when there is a single check-in staff member and more than one infusion nurse.

## Discussion

Based on data collected from three U.S. COVID-19 mAb infusion centers, we conducted simulations allocating personnel and other resources in mAb infusion sites and translated the results into a web-based decision-support tool. The simulation experiment results and our analyses led to several important findings. In particular, despite nursing staff shortages often being perceived as the main barrier, this study identified that other factors were equally important for the overall performance of a mAb site. Frequently, patient LoS was extended due to other factors such as physical capacity, staffing levels at check-in, or the duration of infusion process, even with low nursing levels. These findings suggest that decisions should be made carefully for all key components of a mAb infusion process to provide timely service and improve staffing and capacity efficiency.

Regarding the value of appointment scheduling, it is common to assume scheduling would yield better performance as it allows planning and preparation for upcoming patients beforehand. Yet, this analysis revealed that scheduling appointments was not necessarily beneficial when daily patient volumes were above a level that a mAb site can effectively manage. In those situations, patient load on resources should be reduced by either extending business hours or increasing the levels of the limiting resource.

Achieving a reduction in the duration of a critical service, such as infusion or medication preparation time, did not always lead to significant improvements. In particular, when an infusion site continued to experience high traffic intensity resulting from high patient volumes, short business hours, or low capacity and staffing levels, improvements in patient LoS were minimal. However, when traffic intensity was only slightly above manageable levels, shortening the duration of a medical service (e.g., infusion time) achieved stability and made a significant impact for the operational performance of mAb sites. These results suggest that the infusion sites experiencing patient traffic that is only marginally higher than their physical and staff capacity could benefit the most from shortened service durations, which, for instance, can be achieved by using subcutaneous route for mAb administration instead of IV route.

The demand and need for mAb infusions will vary across communities and over time. Overall, the results from this study suggest that health systems in the U.S. can effectively provide mAb treatments under different settings even with relatively low investments for physical resources and staff. This is not to say that the launch of a mAb site is without barriers, but that this service can be made available to a community and reduce hospitalizations and COVID-19 deaths without a heavy burden on resources. The key, here, is to correctly identify the bottlenecks of the mAb infusion process in the corresponding setting and make the right adjustments to efficiently use limited resources. By analyzing mAb infusions under different conditions and resource allocations, this study can offer guidance for the optimal planning and implementation of mAb infusion sites, which can have a considerable impact in regional COVID-19 response efforts.

This study was not free of limitations. First, the process model aligned with field observations for several sites and was validated by focus groups with those same sites. Hospitals may choose to implement mAb infusions in a different setting. Second, simulations relied on several assumptions for service times, nurse-to-patient ratio in infusion area, and arrival patterns under different scheduling protocols. Differences in these assumptions could impact simulation results. Third, despite considering the majority of the practical scenarios, the web-based decision-support tool is not exhaustive of all possible cases. Accordingly, users will not always be able to select the exact model input values corresponding to the scenario(s) they desire to investigate. Fourth, the data collected from three U.S. COVID-19 mAb infusion centers for our study might not exactly match particular mAb sites especially if patient demographics or geographical conditions are significantly different. Finally, in addition to IV infusions, mAb treatments have also been approved as subcutaneous injections, which were not the focus of our study.

## Conclusion

During his speech addressing the nation on September 9, 2021, President Biden emphasized the importance of mAb treatments for saving lives and reducing the strain on the U.S. healthcare system by preventing severe disease and reducing hospitalizations and reiterated his administration's commitment to making mAb treatments available (18). Following the president's speech, the U.S. government agreed to purchase 1.4 million doses of additional mAb medications casirivimab and imdevimab, as declared by President Biden (18) and announced by Regeneron Pharmaceuticals (19). Concurrently, the Department of Health and Human Services' (HHS) Assistant Secretary for Preparedness and Response (ASPR) took over the distribution of COVID-19 mAb therapies over the country to ensure the availability of this critical COVID-19 treatment across all states and territories (20). Since undertaking the distribution of mAb therapies, ASPR has been in coordination with thousands of individual healthcare facilities and regional leaders to allocate mAb supplies to 4,280 separate healthcare sites throughout the nation (https://protect-public.hhs.gov/pages/therapeutics-distribution), as of October 14 2021, on a weekly basis (12). In parallel with these efforts, the federal government has also initiated to deploy “mAb strike teams” through HHS, Federal Emergency Management Agency, and Department of Defense to support the staffing needs of mAb infusion sites and increase the public access to COVID-19 mAb therapeutics, as declared in Biden Administration's COVID-19 action plan (21). Overall, the active engagement of the federal government and agencies in leading all of these recent efforts and establishing cooperation with local governments and individual healthcare facilities, has been very critical to leverage the maximum use of COVID-19 mAb therapies. These efforts secured the continued availability of mAb injections for the Americans infected with COVID-19, played a fundamental role for achieving an organized and effective implementation of mAb treatments throughout the country, and prevented further burdening of the U.S. healthcare system during its fight against a pandemic. By developing an online decision-support tool for COVID-19 mAb treatment process, we seek to complement these efforts and facilitate complex resource allocation and planning decisions that are needed to be made for each mAb infusion site.

This effort used a simulation approach to create a capacity and staffing planning tool to support adoption and spread of mAb therapy for COVID-19. The objective was to better understand staffing, physical resources, and other requirements for providing timely mAb infusion services with efficient resource use. Considering different staffing and capacity levels, scheduling protocols, patient demand, service hours, and infusion durations, 162,000 scenarios were evaluated via simulation experiments. A web-based decision-support tool was created to allow decision-makers, researchers, and other users to easily access the results, investigate operational performance of mAb infusion sites under different conditions, and generate managerial insights for existing and future infusion sites. Given that mAb treatments are expected to continue being an important instrument for the management of COVID-19, the web-based calculator introduced in this study could significantly contribute to pandemic control, planning, and preparation efforts.

## Data Availability Statement

Data were collected from three monoclonal infusion sites, operating with federal field team support from the U.S. Department of Health and Human Services' Office of the Assistant Secretary for preparedness and response. Requests to access the datasets should be directed to Dr. Çaglar Çaglayan via his email Caglar.Caglayan@jhuapl.edu.

## Author Contributions

ÇÇ and AL designed the conceptual model. ÇÇ performed data and statistical analysis, developed the simulation model, conducted simulation experiments, and wrote the first draft of the manuscript. MS designed and programmed the user interface for the online calculator. ÇÇ and DR performed verification and validation analysis. KR-L performed major edits on the manuscript. JT managed the overall project. TP and JR served as subject-matter experts. JR supervised the project and provided mentorship. All authors contributed to conception and design of the study, contributed to manuscript revision, read, and approved the submitted version.

## Funding

This study was supported by the U.S. Department of Health and Human Services' Office of the Assistant Secretary for Preparedness and Response. Virtual machines belonging to the Johns Hopkins University Applied Physics Laboratory were utilized to perform the simulation experiments. The findings and conclusions in this paper are those of the authors and do not represent the official position of any government or private organization.

## Conflict of Interest

The authors declare that the research was conducted in the absence of any commercial or financial relationships that could be construed as a potential conflict of interest.

## Publisher's Note

All claims expressed in this article are solely those of the authors and do not necessarily represent those of their affiliated organizations, or those of the publisher, the editors and the reviewers. Any product that may be evaluated in this article, or claim that may be made by its manufacturer, is not guaranteed or endorsed by the publisher.
