# Supplementary material for: Staffing and Capacity Planning for SARS-CoV-2 Monoclonal Antibody Infusion Facilities: A Performance Estimation Calculator Based on Discrete-Event Simulations
Source: Front Public Health. 2022 Jan 28;9:770039. doi: 10.3389/fpubh.2021.770039 (PMC8831825; doi:10.3389/fpubh.2021.770039)
Supplement: Supplementary file 1 [file Data_Sheet_1.pdf]

Supplemental Material

Appendix A – Data and Description for Generating Patient Arrival Times and Adverse Events

For walk-in appointments, we took a two-step approach to generate patient arrivals. First, utilizing an emergency department data from a large-scale hospital, we derived an empirical probability density function for the number of arrivals, measuring the likelihood of a patient arrival for each one-hour interval during a day. Next, for a given total number of arrivals pre-specified by the scenario to be simulated, we used this empirical probability density function to determine how many of these patients to arrive at each one-hour slot during the service hours. Once the number of arriving patients for each one-hour interval was determined, we then employed a uniform probability distribution for every one-hour interval to generate the arrival times of the walk-in patients. For scheduled appointments, appointment times were determined by the scheduling type. Yet, we also adjusted the patient arrival times around pre-scheduled appointment times by using normally distributed a random variable to account for delays and early arrivals. The probability distributions and parameters for walk-in arrivals and late and early arrival adjustments for scheduled arrivals are provided in **Table A1**.

Process	Probability Distribution	Parameters (Minutes)
Early or Late Arrival to Scheduled Appointments	Normal	Mean = 7.5,<br>Standard Deviation = 2
Walk-in Arrival	Empirical	Prob. = 0.0517 for 9.00 - 9.59 am<br>Prob. = 0.0623 for 10.00 - 10.59 am<br>Prob. = 0.0617 for 11.00 - 11.59 am<br>Prob. = 0.0613 for 12.00 - 12.59 pm<br>Prob. = 0.0576 for 1.00 - 1.59 pm<br>Prob. = 0.0564 for 2.00 - 2.59 pm<br>Prob. = 0.0565 for 3.00 - 3.59 pm<br>Prob. = 0.0561 for 4.00 - 4.59 pm<br>Prob. = 0.0560 for 5.00 - 5.59 pm<br>Prob. = 0.0560 for 6.00 - 6.59 pm<br>Prob. = 0.0574 for 7.00 - 7.59 pm<br>Prob. = 0.0514 for 8.00 - 8.59 pm<br>Prob. = 0.0464 for 9.00 - 9.59 pm<br>Prob. = 0.0397 for 10.00 - 10.59 pm<br>Prob. = 0.0287 for 11.00 - 11.59 pm<br>Prob. = 0.0277 for 12.00 - 12.59 am<br>Prob. = 0.0217 for 1.00 - 1.59 am<br>Prob. = 0.0185 for 2.00 - 2.59 am<br>Prob. = 0.0175 for 3.00 - 3.59 am<br>Prob. = 0.0160 for 4.00 - 4.59 am<br>Prob. = 0.0159 for 5.00 - 5.59 am<br>Prob. = 0.0187 for 6.00 - 6.59 am<br>Prob. = 0.0248 for 7.00 - 7.59 am<br>Prob. = 0.0400 for 8.00 - 8.59 am

Table A1. Parameters for Early or Late Arrivals and Probability Function for Walk-in Arrivals

Both for walk-in and for scheduled appointments, occasionally, we observed prolonged post-infusion observation times. Despite being rare, the delay resulted from several reasons including concerns about the observed patient's health status, medical equipment failure, or minor adverse events such as bleeding after IV catheter removal. The frequency of a prolonged observation was 5% and the duration of an increase in post-infusion observation service was 15, 30, 45, or 60 minutes with probabilities 0.50, 0.25, 0.20, and 0.05, respectively (See **Table A2**).

Process	Probability Distribution	Parameters (Minutes)
Prolonged Observation	Discrete	Probability of Occurrence = 0.05<br>Duration of Delay = 15, 30, 45, or 60<br>Duration Prob. = 0.50, 0.25, 0.20, 0.05

Table A2. Parameters for Prolonged Post-Infusion Observation Process

Appendix B - The Simulation Events and Main Steps of The Simulation Algorithm

From patient arrival to departure, the overall mAb treatment process typically consists of pre-infusion check-in, chair placement and IV catheter insertion, infusion, post-infusion observation, and discharge steps. For walk-in appointments, these processes are accompanied by medication preparation process, performed either on-site or at an external pharmacy, whereas medication preparation is assumed to be completed in advance for scheduled appointments. Among these activities, the discrete-event simulation algorithm identifies the next event at each iteration, performs system status updates corresponding the next simulation event, advances the simulation clock, and then performs the next iteration by repeating this simulation logic until the closure time of the mAb facility. The events that are modeled and simulated by the discrete-event simulations are briefly described as follows:

- Patient Arrival: In the check-in area, the patients are served based on first-come-first-served rule. Accordingly, a newly arrived patient begins her check-in process if there is an idle staff member in the check-in area. Otherwise, she joins the queue and waits for her service either in the check-in area or outside (e.g., in her car).

- Check-in Process Completion: Following the completion of the pre-infusion check-in service, each patient is admitted to the infusion area given the physical capacity permits and the infusion area clinical staff, consisting of nurses and allied health professionals (e.g., paramedics), are available to initiate her bed/chair placement and IV catheter insertion process. Yet, when all of the treatment beds/chairs are occupied or the clinical personnel in the infusion area are busy with serving other patients, patients continue to stay in the check-in area and wait. Meanwhile, the check-in process of another patient can be initiated.

- Chair/Bed Placement and IV Catheter Insertion Completion: Once a patient is placed on a treatment bed/chair and her IV catheter is inserted, the patient becomes ready for her mAb infusion therapy given her medication is also available. At this point, the medical team decides whether to

initiate the infusion process of this patient or bed/chair placement and IV catheter insertion of another patient depending on the longest waiting line. Note that infusion process cannot begin if there is no medication available and similarly, bed/chair placement process cannot be initiated if there is no idle treatment bed/chair and available medical personnel. When neither process can be initiated, a member of the infusion team performs discharge tasks for one of the patients waiting for their discharge process to be completed before their departure.

- Infusion Process Completion: The infusion process is automated, takes a fixed amount of time (e.g., 20, 30, or 60 minutes) depending on the medication, and is performed under the supervision of the infusion area medical team. Once it is completed, the one-hour long observation process begins for the patient. At the same time, the infusion team begins to discharge a patient waiting for her departure or initiates bed/chair placement or infusion process of another patient depending on the queue length and availability of a treatment bed/chair or infusion medication.

- Observation Completion: Following the post-infusion observation process, a patient joins the discharge process queue and begins waiting for his discharge procedure to be initiated. Meanwhile, the infusion team initiates the discharge process for the head of the waiting line patient and continue to supervise other patients in the infusion area.

- Discharge Completion: Discharge is the last process of the overall mAb infusion therapy, following which patients depart the mAb infusion center. Accordingly, after each discharge completion, a treatment bed/chair becomes available for the use of another patient. The medical team in the infusion area either admits a new patient to the infusion area by starting that patient's bed/chair placement process or initiates the infusion process for a different patient. This choice is made based on the largest waiting queue length between bed/chair placement and infusion processes, where the lack of medication to be administered delays the infusion process.

- Medication Preparation Completion: For scheduled appointments, the medication preparation is assumed to be performed in advance. For walk-in appointments, the completion of the check-in process initiates medication preparation either on-site or at an external pharmacy. Once a medication is prepared, it is sent to the infusion area and becomes available to be administered. If there is a patient waiting for her infusion process due to the lack of a medication, the newly prepared medication enables the initiation of the infusion process for this patient.

Appendix C - The Structure of The Discrete-Event Simulation Model

The programming code for the discrete-event simulation model consists of two major parts: the main code and simulation function. By retrieving model inputs an excel document, the main code specifies the values of the model variables for the scenario of interest to be simulated such as the number of treatment beds, appointment type, and facility service hours. It also defines the probability distributions and parameters for the stochastic and deterministic processes (e.g., exponential distribution with mean 20 minutes for check-in process) and uses them to execute random number generations for every process from patient arrival to departure. Then, the main code forwards these inputs to the simulation

function, commands it to run the scenario of interest with a pre-determined number of replications, records the summary statistics with performance measures, and reports the model outputs to the user for the evaluated scenario.

Discrete-event simulation experiments are performed by the second component of the programming code: the simulation function. A fixed number of replications are independently run by the simulation function for all of the scenarios to be simulated. For each replication of a scenario, the simulation code initiates its execution at the opening time of the mAb infusion site. Then, the code identifies the next event, updates the system status depending on the (changes the) next event (causes), collects statistics, and forwards the simulation clock to the time of the next event in this respective order. This process sequence is iterated by the simulation function until the closure time of the mAb infusion site, which marks the termination time of the current replication. Subsequently, the simulation function moves forward to running the next replication for the same scenario and reports the summary statistics together with performance outputs to the main code when all of the replications are completed for the given scenario.

Employing the discrete-event simulation model and utilizing the scheme described in this paper, 162,000 different scenarios were assessed by simulation experiments. The number of scenarios corresponding to different infusion durations and scheduling types are reported in **Table C**.

**Infusion Duration**	**Total Number of Scenarios**	**Scheduling Type**	**Number of Scenarios**
20 minutes	54,000	Walk-in	13,500
		Spread-Out	13,500
		Mixed	13,500
		Block	13,500
30 minutes	54,000	Walk-in	13,500
		Spread-Out	13,500
		Mixed	13,500
		Block	13,500
60 minutes	54,000	Walk-in	13,500
		Spread-Out	13,500
		Mixed	13,500
		Block	13,500

Table C. The Distribution of Simulated Scenarios

Appendix D – An Example of System Performance Analysis Based on Univariate Changes

Here, we present an example, demonstrating how users can manually assess the impact of changing the value of particular factors on the system performance metrics for a given scenario of interest. To do so, we arbitrarily chose a base-case scenario, and changed the value of a single feature while keeping all other factors the same. The reference scenario corresponded to an infusion site with the following features: (i) 6 treatment beds/chairs, (ii) scheduled arrivals with spread-out appointments, (iii) 10-hour long daily service, (iv) 20 patients seeking service per day on average, (v) 2 check-in area personnel, (vi) 60-minute infusions per patient, (vii) 1 infusion nurse, and (viii) no allied health professional supporting the infusion area. Using this base-case scenario (**Figure D Upper Left**), we evaluated three alternative cases for the infusion site, where the first case had 20 beds/chairs instead of 6 (**Figure D Upper Right**), the second case had 1 non-nurse infusion staff instead of none (**Figure D Lower Left**), and the third case had 30-minute-long infusions instead of 60 minutes (**Figure D Lower Right**). This analysis revealed that, for the particular scenario, increasing the staffing levels in the infusion area or decreasing the duration of the infusion treatment significantly improved the performance metrics such as the number of patients that can be seen, average treatment duration, etc., whereas increasing the number of treatment beds while keeping other factors unchanged had almost no effect.

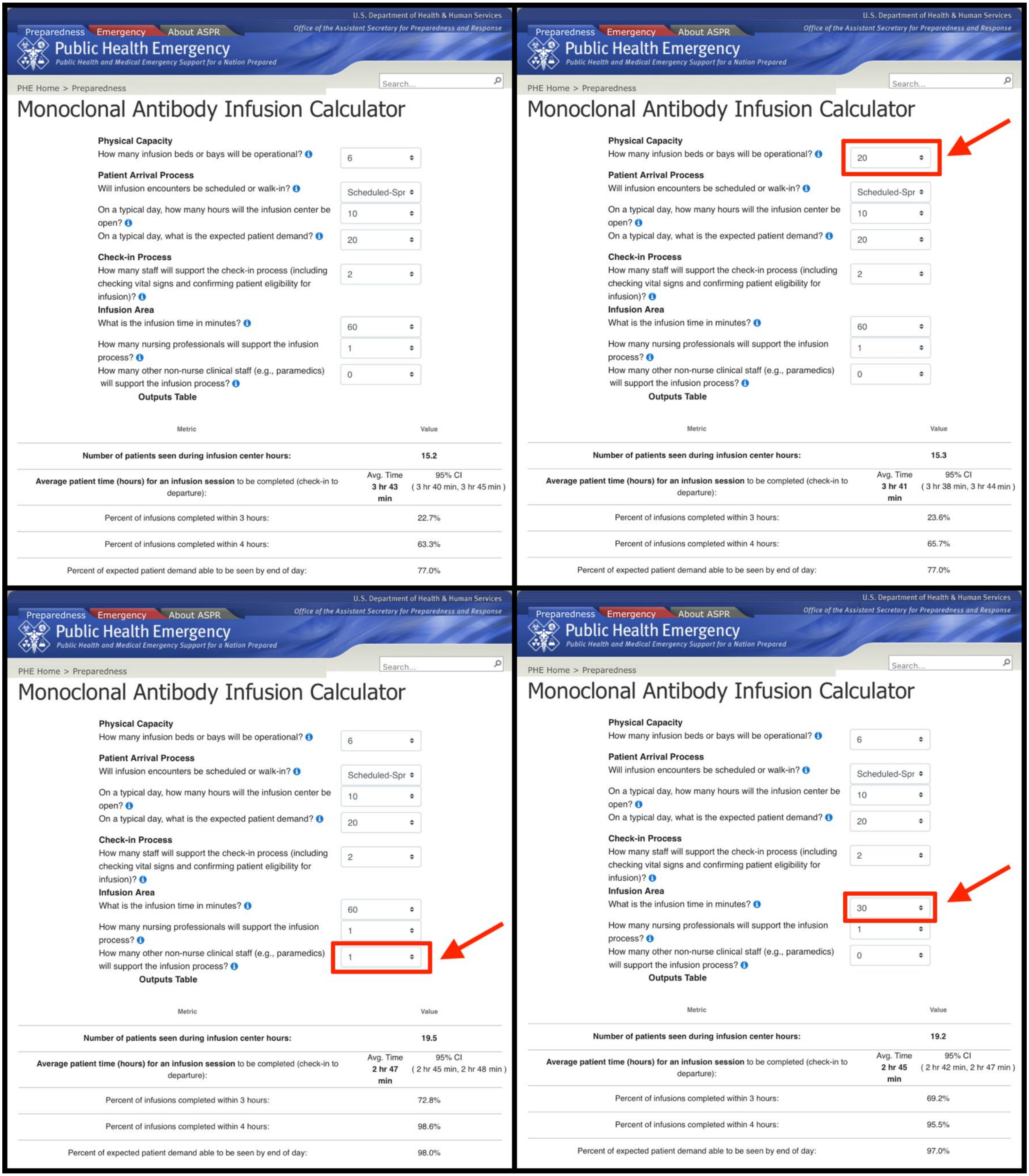

Figure D. Parameters for Early or Late Arrivals and Probability Function for Walk-in Arrivals
